# Toxicity and Lethal Effect of Greenhouse Insecticides on *Coccinella septempunctata* (Coleoptera: Coccinellidae) as Biological Control Agent of *Myzus persicae* (Hemiptera: Aphididae)

**DOI:** 10.3390/toxics11070584

**Published:** 2023-07-05

**Authors:** Panagiotis J. Skouras, Eirini Karanastasi, Ioannis Lycoskoufis, Vasilis Demopoulos, Anastasios I. Darras, Athanasios Tsafouros, Polina C. Tsalgatidou, John T. Margaritopoulos, George J. Stathas

**Affiliations:** 1 Laboratory of Agricultural Entomology and Zoology, Department of Agriculture, University of the Peloponnese, Kalamata Campus, 24100 Antikalamos, Greece; g.stathas@uop.gr; 2 Laboratory of Plant Protection, Department of Agriculture, University of the Peloponnese, Kalamata Campus, 24100 Antikalamos, Greece; v.dimopoulos@go.uop.gr; 3 Plant Protection Laboratory, Department of Agriculture, University of Patras, Nea Ktiria, 30200 Mesolonghi, Greece; ekaranastasi@upatras.gr; 4 Department of Agriculture, University of the Peloponnese, 24100 Kalamata, Greece; i.lykoskoufis@uop.gr (I.L.); a.darras@uop.gr (A.I.D.); thantsaf@hotmail.com (A.T.); polina.tsalgatidou@go.uop.gr (P.C.T.); 5 Department of Plant Protection, Institute of Industrial and Fodder Crops, Hellenic Agricultural Organization “DEMETER”, 38334 Volos, Greece; johnmargaritopoulos@elgo.com

**Keywords:** biological control, ecotoxicology, greenhouse, insecticides, IPM, side effect, coccinellids

## Abstract

Deltamethrin and imidacloprid are commonly used insecticides for controlling sub-sucking insects in greenhouses. However, their application may cause sublethal effects on the aphid coccinellid predator *Coccinella septempunctata* (Coleoptera: Coccinellidae). Here, we study (i) the toxicity and the effect of two sublethal doses (LD_10_ and LD_30_) of deltamethrin and imidacloprid on *C. septempunctata* in a laboratory microcosm and (ii) the residual toxicity of the two insecticides in a greenhouse. The results showed that both insecticides reduced fecundity, longevity, the intrinsic rate of increase, the finite rate of increase and the net reproductive rate. However, the developmental time of the fourth instar larvae was prolonged by both insecticides at LD_10_ and LD_30_. Deltamethrin residues were toxic 21 DAT (days after treatment) to *C. septempunctata* fourth instar larvae. In contrast, imidacloprid began in the slightly harmful category (75%) 1 DAT and declined to the harmless category (18.33%) 21 DAT. These results indicate that deltamethrin and imidacloprid have potential risks to *C. septempunctata*. This study provides information to guide the development of integrated pest management (IPM) strategies in greenhouses.

## 1. Introduction

Aphids (Hemiptera: Aphididae) comprise an insect group that feeds on a wide range of plant species. In greenhouse crops, aphids are amongst the most dominant and destructive pests, causing significant losses in quality and/or yield [1]. The green peach aphid, *Myzus persicae* (Sulzer) (Hemiptera: Aphididae), is extremely polyphagous, with great efficiency as a virus vector [2]. *Myzus persicae* can seriously damage crops by feeding on the vascular bundles of plants and/or by transmitting more than 100 plant viruses [3]. Chemical insecticides are widely used to control aphids and especially *M. persicae* [4]. However, long-term exposure of agricultural systems to insecticides has led to the development of resistance to many classes of insecticides, including pyrethroids and neonicotinoids [5,6]. Concerns about insecticide resistance development and the rapid emergence of insecticide resistance of *M. persicae* populations to new active ingredients [7] or insecticides with different MoA have increased the interest in integrated pest management (IPM) adoption for aphid control [4,6,8].

Biological control of aphids with parasitoids and/or predators is a critical component of IPM programs. Coccinellid predators are often utilized in greenhouses for aphid management [9], since many species can reduce aphid populations in greenhouses [10,11]. Coccinellid predator release can reduce the aphid population and is considered an alternative to insecticide applications [9,12].

The seven-spotted lady beetle, *Coccinella septempunctata* L. (Coleoptera: Coccinellidae), is an excellent biological control agent because it preys on more than 20 aphid species of Coccoidea, as well as species of Psylloidea and Tetranychidae, that infest crops both in the fields and in greenhouses [13] and can be found in a wide range of agricultural and/or natural habitats or crops all over the world [14]. Larvae and adults of *C. septempunctata* preying on aphids pose a major advantage since they could prevent aphid density increases from reaching the economic thresholds (ETs). However, when aphid population density exceeds the ET, the use of selective pesticides is critical for preserving *C. septempunctata* populations and achieving successful aphid predation rates [15].

Two of the most commonly used classes of insecticides applied to control sap-sucking pests in greenhouses and fields are neonicotinoids and pyrethroid [4,16]. However, in the European Union (EU), the neonicotinoids, imidacloprid, clothianidin and thiamethoxam in 2017 and thiacloprid in 2020 were banned for all outdoor uses [17,18] due to chronic adverse effects on honeybees [19]. In the present study, we aimed to evaluate the toxicity, developmental time, survival and adults’ longevity by examining the pre-oviposition period (APOP), total pre-oviposition period (TPOP), fecundity and population growth parameters of *C. septempunctata* exposed to lethal and sublethal doses of imidacloprid and deltamethrin. Additionally, in this study, we tried to examine this assumption of differential mortality by directly assessing the residual toxicity of imidacloprid and deltamethrin of *C. septempunctata* in the greenhouse. The present findings could assist the conservation of *C. septempunctata* and the regulation of deltamethrin and imidacloprid in IPM programs in greenhouses.

## 2. Materials and Methods

### 2.1. Insect Rearing

In total, more than 200 adults of *C. septempunctata* were collected from tobacco and peach fields infested by *M. persicae* in Katerini, Greece, and transferred to the Laboratory of Agricultural Entomology & Zoology of the University of the Peloponnese (Kalamata City, Greece). The green peach aphid, *M. persicae,* was used to rear *C. septempunctata*. Laboratory cultures of *C. septempunctata* were held on Chinese cabbage *Brassica rapa pekinensis* Hanelt (Brassicaceae) plants in cylindrical acrylic glass cages (DL: 30 × 50 cm) at 25 ± 1 °C and a relative humidity (RH) of 60–70% with a photoperiod of 16:8 h light: darkness (L:D).

### 2.2. Insecticides

The experiments were conducted with two commercial insecticides, imidacloprid (Confidor Forte 200SL, Bayer CropScience Hellas, Marousi, Greece) and deltamethrin (Decis 25EC, Bayer CropScience Hellas, Marousi, Greece).

### 2.3. Dose Response to Topical Application Bioassays

Independent assays were performed to find the acute toxicity of deltamethrin and imidacloprid against 4th instar larvae of *C. septempunctata*. The assays were carried out by topical application following a modified protocol of the method of [20,21]. Imidacloprid was dissolved in acetone to prepare a concentration gradient of 100, 200, 400, 600, 800, 1000, 1200 and 1600 ng of active ingredient per insect. The corresponding values for deltamethrin were 0.20, 0.40, 0.80, 1.60, 3.20, 4.80 and 6.40 ng of active ingredient per insect. Fourth instar larvae of *C. septempunctata* (<24 h old) were transferred to a Blackman box [22], and 1 μL of insecticide at each concentration was applied to the mesonotum of *C. septempunctata* using a 10 μL Hamilton microsyringe. Larvae treated with 1 μL acetone alone were set as control. Each larva was placed separately in a Blackman box with more than five hundred live *M. persicae* aphids. There were seventeen treatments including control, and every treatment was repeated three times using twenty larvae per treatment. Treated larvae were maintained in a controlled environmental chamber at 23 ± 1 °C, L16:D8 and 50 ± 5% RH. Mortality data for *C. septempunctata* were scored after 72 h. Larvae were considered dead if they did not move when gently pushed by a fine brush.

### 2.4. Evaluation of Low and Sublethal Effects on Fourth Instar Larvae

To calculate the life table parameters for *C. septempunctata*, a total of 361 eggs (12–24 h old) were randomly collected and maintained in Petri dishes (9 cm diameter). We used 65 eggs for the control group, 70 and 75 eggs in the deltamethrin LD_10_ and LD_30_ groups, respectively, and 71 and 80 eggs in the imidacloprid LD_10_ and LD_30_ groups, respectively. The hatch rate and incubation period of *C. septempunctata* eggs were recorded daily. After egg hatching, each first instar larva was transferred individually into a Blackman box, at the base of which there was a piece of water-saturated moss. Mortality and development time were recorded daily until the fourth instar larvae. To assess the low and sublethal effects on *C. septempunctata* population parameters, fourth instar larvae (<24 h old) topically treated (as described in the dose response to topical application bioassays section) to LD_10_ and LD_30_ doses of deltamethrin (0.34 ng a.i. and 0.63 ng a.i. per insect, respectively) and to LD_10_ and LD_30_ doses of imidacloprid (357.96 ng a.i. and 519.13 ng a.i. per insect, respectively), which were calculated from the toxicity regression equation, and as a control, we used only acetone (65 eggs). One fourth instar larva was released in each Blackman box. Larvae were reared and treated as noted in the topical bioassays section. After exposure to the insecticide, development time and mortality were recorded daily until the emergence of adults. After eclosion, adult females and males of each dose were randomly paired and transferred to a new Blackman box. Each pair was fed ad libitum with *M. persicae* every 24 h. The survival rate and fecundity were scored daily until all adults were dead. All insects were maintained in a controlled environmental chamber at 23 ± 1 °C, L16:D8 and 50 ± 5% RH.

### 2.5. Greenhouse Residual Toxicity Test

Seven hundred and twenty plants of *Capsicum annuum* L. were maintained in a greenhouse at 20 ± 3 °C with additional light (16 h light: 8 h dark) and were grown individually (Supplementary File). Plants at the five-leaf stage were used for the experiment. Pepper plants were sprayed (at the highest dose recommended on the label) until run off with deltamethrin (17.5 mg a.i L^−1^) or imidacloprid (60 mg a.i L^−1^) or tap water for control plants using a manual backpack sprayer. Bioassays on pepper plants 1, 3, 10 and 21 days after insecticide application (DAT) were conducted by placing fourth instar larvae (<24 h old) of *C. septempunctata*. In addition, about more than 300 frozen *M. persicae* aphids were placed on each plant to assure that all larvae had access to food during the experiment, and each plant–larvae system was covered by muslin to avoid insects’ escape. Each plant counted as a replicate. A total of sixty larvae per treatment were used. Larvae mortality was recorded 3 and 7 days after exposure.

### 2.6. Statistical Analysis

The predator dose–mortality relationship of LD_50_ and sublethal (LD_10_ and LD_30_), doses of the insecticides, 95% confidence intervals (CI), slopes and chi-square were calculated by probit analysis using SPSS version 25.0 (SPSS Inc., Chicago, IL, USA). The software two-sex MSChart [23] was used to analyze the life history data of *C. septempunctata* after exposure to sublethal doses of the insecticides imidacloprid and deltamethrin, based on the age-stage two-sex life table theory [24,25]. The life table parameters (*l_x_*), (*m_x_*), (*e_xj_*), (*v_xj_*) and (*s_xj_*) (age-specific survival rate, age-specific fecundity, age-stage life expectancy, age-stage reproductive value and age-stage survival rate, respectively) were calculated. The pre-adult developmental duration time, pre-oviposition and total pre-oviposition period (APOP and TPOP, respectively) fecundity, male and female duration time, intrinsic rate of increase (*r*), finite rate of increase (*λ*), net reproductive rate (*R*_0_) and mean generation time (*T*) were also calculated. The paired bootstrap test was used to analyze the difference among each treatment group for all population parameters [26].

The residual effect of each insecticide on *C. septempunctata* mortality was compared using the χ^2^ test. When χ^2^ was significant, pairwise comparisons were performed using the Bonferroni correction.

## 3. Results

### 3.1. Toxicity of Deltamethrin and Imidacloprid to C. septempunctata

The toxicity of deltamethrin and imidacloprid against fourth instar larvae of *C. septempunctata* was determined after 3 days (Table 1). The LD_50_, LD_10_ and LD_30_ values of deltamethrin were 0.98, 0.337 and 0.633 ng a.i. per insect, respectively. The corresponding values in the case of imidacloprid were 671.56, 519.13 and 357.96 ng a.i per insect, respectively. The LD_10_ and LD_30_ values of deltamethrin and imidacloprid obtained were used to calculate the sublethal effects of both insecticides on the population parameters of *C. septempunctata*.

### 3.2. Sublethal Effects of Deltamethrin and Imidacloprid on C. septempunctata

The developmental time of fourth instar larvae of *C. septempunctata* at all doses was significantly longer than that of the control group (Table 2). There were no significant differences between the deltamethrin and imidacloprid LD_10_ and LD_30_ groups. Furthermore, the developmental time of pupa did not differ in deltamethrin LD_30_, two imidacloprid and the control groups, while deltamethrin LD_10_ resulted in a significantly lower developmental time (4.64 days) than the control group.

Adult longevity was significantly shorter in imidacloprid and deltamethrin treatments than in the control treatment (Table 3). There were no significant differences between imidacloprid and deltamethrin treatments in terms of female adult longevity. In addition, female adult longevity was significantly shorter in the imidacloprid treatments compared with the control treatment. Male adult longevity did not differ significantly between imidacloprid LD_10_ (61.23 days) and the control treatment (70.11 days). Male adult longevity was significantly lower in the treatments of deltamethrin LD_30_ and LD_10_ (52.67 and 50.79 days, respectively) and imidacloprid LD_30_ (50.33 days) compared to the control treatment (70.11 days). No significant differences were recorded between the imidacloprid, deltamethrin and control group in terms of TPOP and APOP (Table 3). The number of eggs laid by females of *C. septempunctata* decreased by 52.07% and 69.89% in imidacloprid LD_10_ and LD_30_ doses, respectively, and by 57.53% and 31.72% in deltamethrin LD_10_ and LD_30_ doses, respectively, when compared with the control treatment. Furthermore, female fecundity did not differ significantly between the deltamethrin LD_30_ treatment and the control treatment, while imidacloprid treatments (LD_10_ and LD_30_ doses) and deltamethrin (LD_10_ dose) resulted in significantly reduced fecundity compared to the control treatment.

The population growth parameters are shown in Table 4. Treatment with imidacloprid had a significant effect on the finite rate of increase (*λ*) and intrinsic rate of increase (*r*) compared to those in the control group. Treatment with deltamethrin and imidacloprid resulted in a significantly smaller net reproductive rate (*R*_0_) in comparison to the control group. However, the mean generation time (*T*) had no significant effect between treatments.

**Figure 1:**
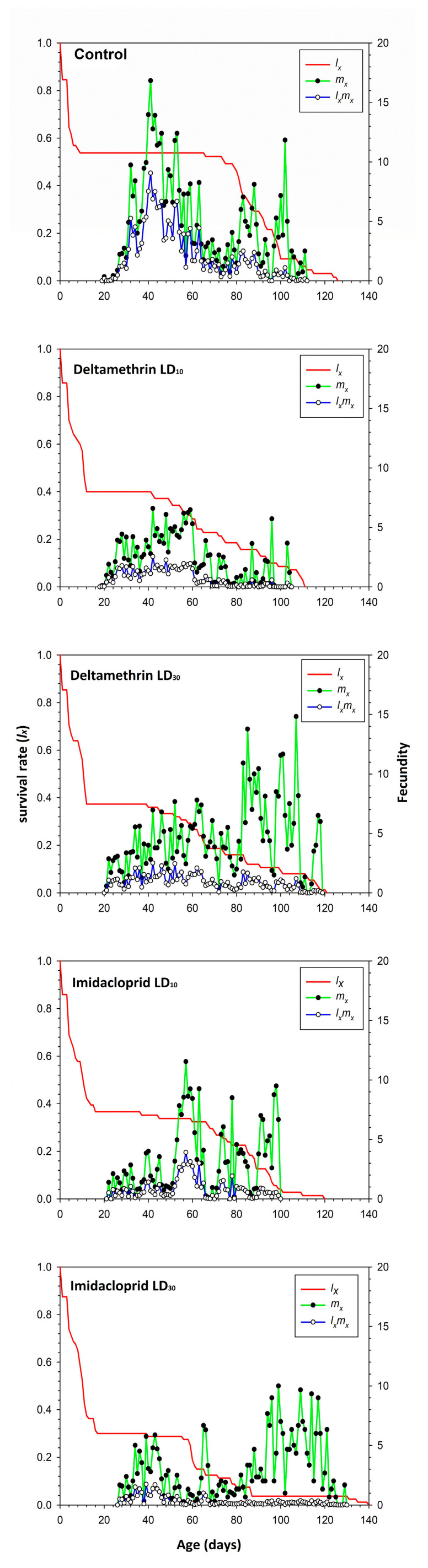
Age-specific survival rate (*l_x_*), age-specific fecundity (*m_x_*), and age-specific maternity (*l_x_m_x_*) after fourth instar *C. septempunctata* larvae exposed to sublethal deltamethrin and imidacloprid doses.

Figure 1 presents the fecundity of the total population (*m_x_*), age-specific survival rate (*l_x_*) and the net maternity (*l_x_m_x_*) of *C. septempunctata*. The analysis of the age-specific survival rate, *l_x_*, demonstrates a more rapid decrease in the deltamethrin and imidacloprid treatment groups than in the control group. The highest *m_x_* (16.8) and *l_x_m_x_* (9.1) in the control group occurred on day 41. In comparison, the lowest *m_x_* (6.6) peak occurred at age 42 days in the deltamethrin LD_10_ group, while the *l_x_m_x_* (1.7) peak was recorded at 43 days in the imidacloprid LD_30_ treatment group.

**Figure 2:**
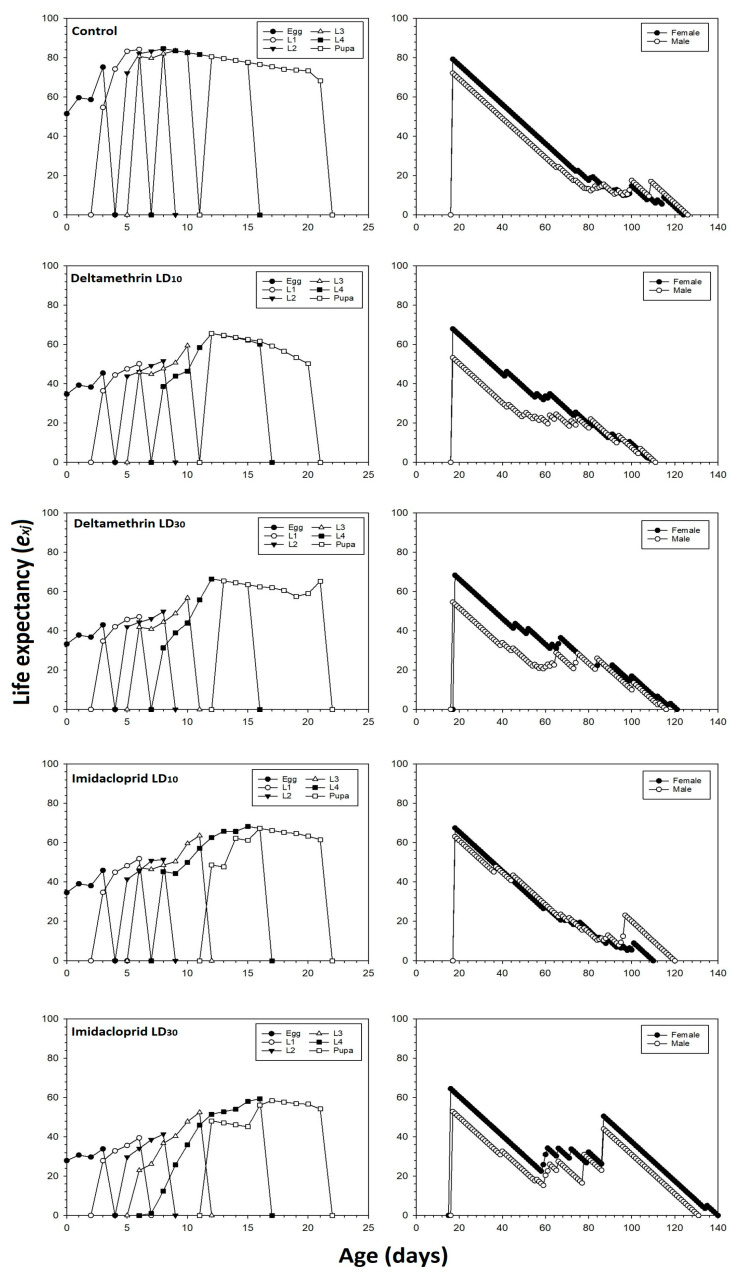
Life expectancy (*e_xj_*) values after fourth instar *C. septempunctata* larvae exposed to sublethal deltamethrin and imidacloprid doses.

The age-stage life expectancy *e_xj_* of *C. septempunctata* is shown in Figure 2. All individuals treated by imidacloprid or deltamethrin have a lower *e_xj_* than the control group. For example, a newly hatched *C. septempunctata* egg was supposed to survive 51.67 days in the control group compared to the life expectancies in deltamethrin LD_10_ and LD_30_ groups, which were 34.69 and 33.28 days, respectively. The corresponding values for imidacloprid were 34.55 and 27.86 days in the LD_10_ and LD_30_ treatments, respectively.

**Figure 3:**
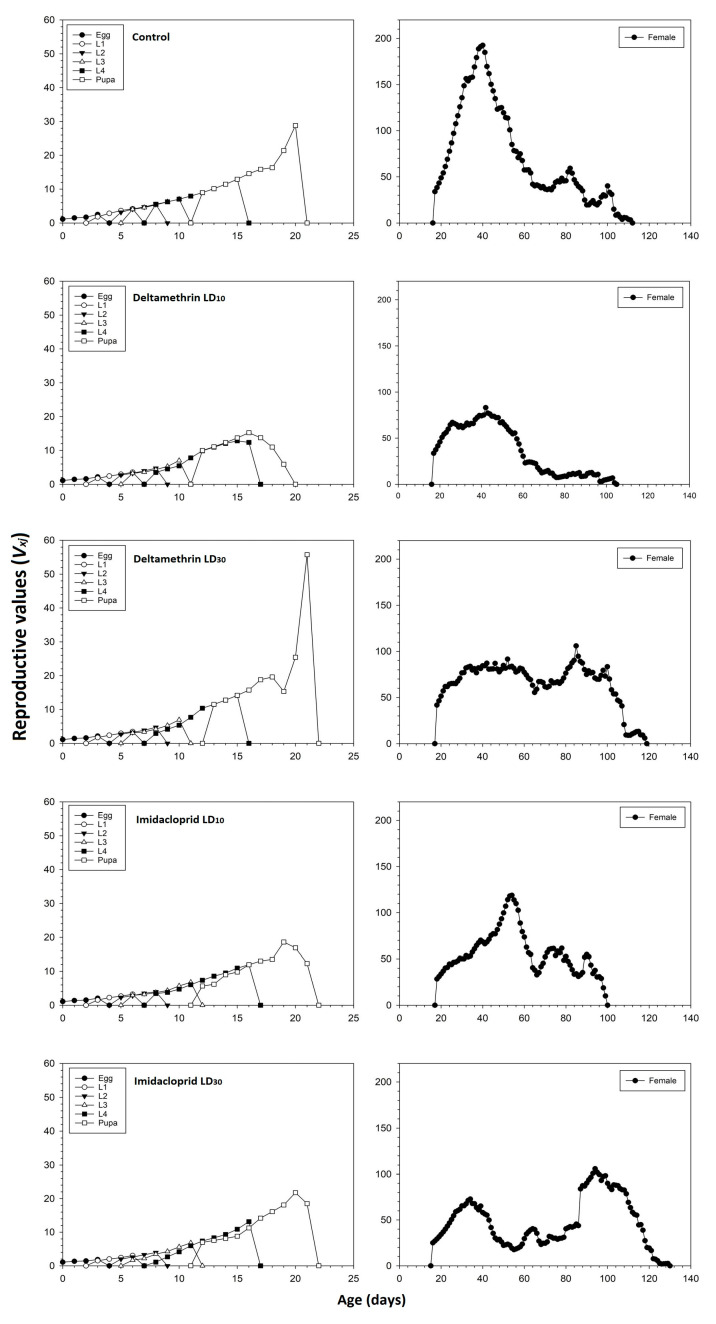
Age-stage specific reproductive values (*V_xj_*) values after fourth instar *C. septempunctata* larvae exposed to sublethal deltamethrin and imidacloprid doses.

The age-stage reproductive value (*V_xj_*) of newly hatched *C. septempunctata* eggs was significantly lower in the imidacloprid LD_10_ and LD_30_ treatments compared to the control and deltamethrin groups (Figure 3). The *V_xj_* began to increase when females started to produce offspring. The peak *V_xj_* value of the untreated control *C. septempunctata* was 192.58 days^−1^ at 40 days. In the deltamethrin LD_10_ and LD_30_ groups, the peak *V_xj_* values were 83.08 and 105.95 days^−1^ found at 42 and 85 days, respectively. In the imidacloprid LD_10_ and LD_30_ groups, the corresponding values were 118.80 and 106.01 days^−1^ found at 54 and 94 days, respectively.

**Figure 4:**
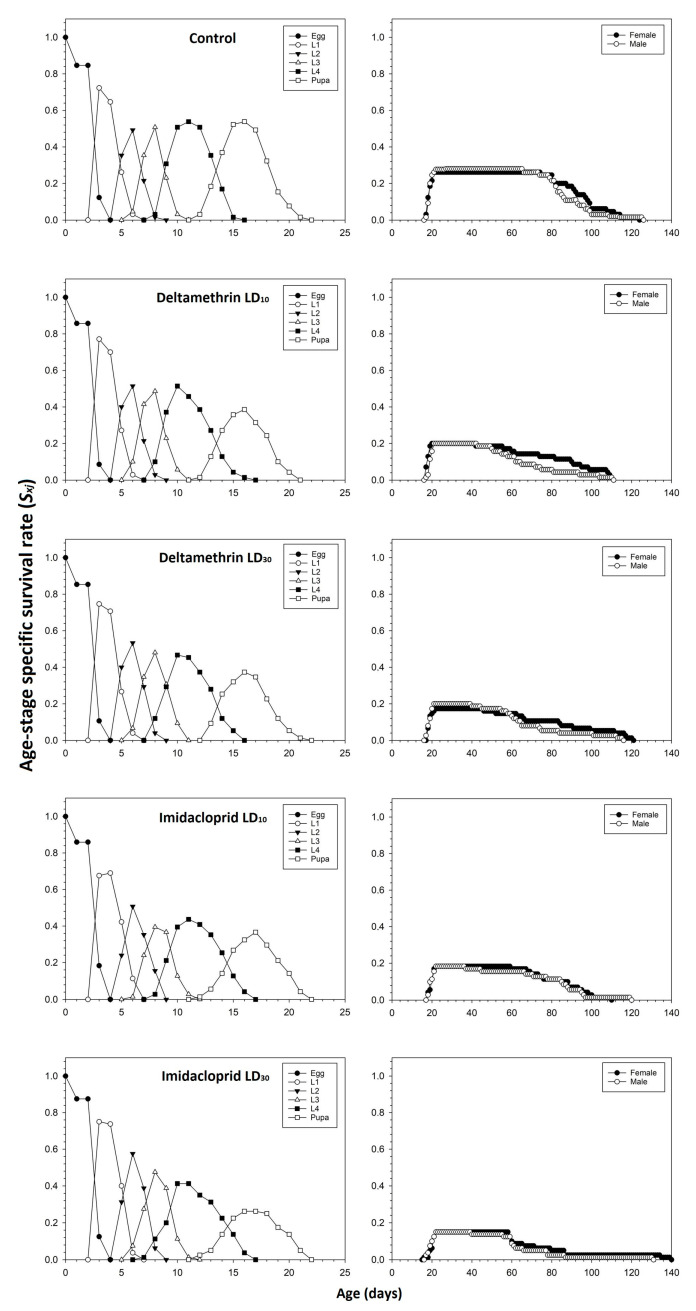
Age-stage-specific survival rate (*S_xj_*) after fourth instar *C. septempunctata* larvae exposed to sublethal deltamethrin and imidacloprid doses.

The age-stage survival rates (*S_xj_*) of *C. septempunctata* were negatively affected in the deltamethrin and imidacloprid treatment groups compared to the control group (Figure 4). The peak *S_xj_* values for male and female adults in the control group were 0.28 and 0.26, respectively. The peak *S_xj_* values for male and female adults treated with deltamethrin (i.e., LD_10_: 0.20 for males and females; LD_30_: 0.20 for males and 0.17 for females) and imidacloprid (i.e., LD_10_: 0.18 for males and females; LD_30_: 0.15 for males and females) decreased by increasing the insecticide doses.

**Figure 5:**
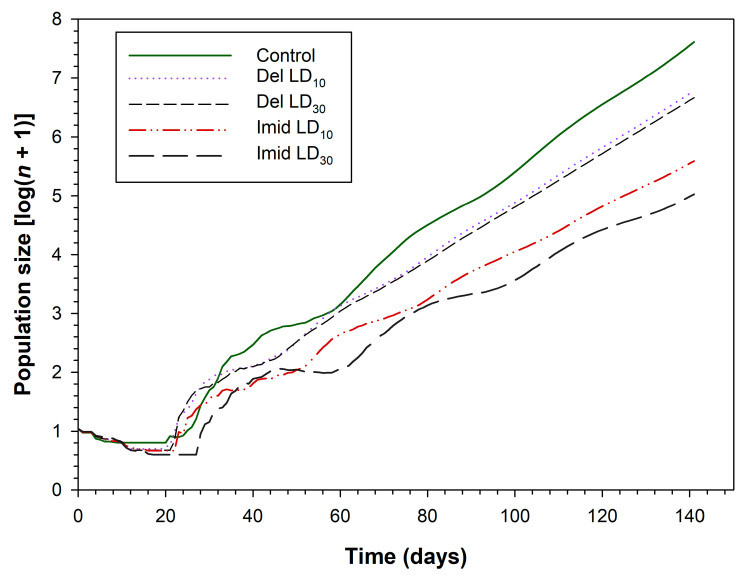
Population projection for *C. septempunctata* larvae exposed to LD_10_ and LD_30_ doses of deltamethrin and imidacloprid.

The projection population size of *C. septempunctata* at 140 days following different insecticide treatments is shown in Figure 5. The population size of *C. septempunctata* after 140 days in the control group was projected to be 7.6-fold greater than the initial population. The corresponding values for deltamethrin were 6.8 and 6.7 in the LD_10_ and LD_30_ groups, respectively. Population size was also affected by imidacloprid at 140 days in the LD_10_ (5.6-fold) and LD_30_ (5.0-fold) groups.

### 3.3. Greenhouse Residual Toxicity Test

**Figure 6:**
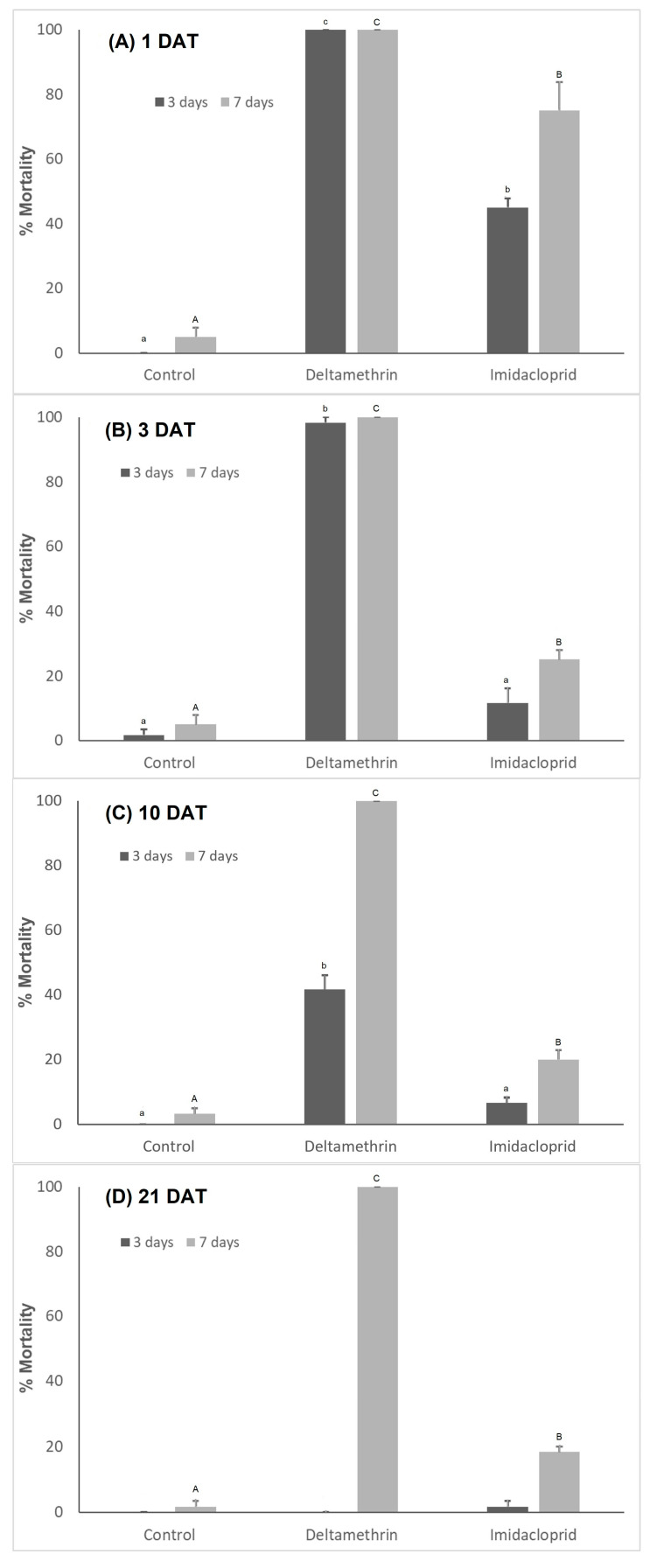
Mean value (±SE) of mortality of *Coccinella septempunctata* larvae when exposed to pepper plants previously sprayed with insecticides for 3 and 7 days. Residues were assayed (**A**) 1 DAT, (**B**) 3 DAT, (**C**) 10 DAT and (**D**) 21 DAT. Means showed by the same letters are not significantly different (*p* < 0.05) according to Duncan test.

The insecticides deltamethrin and imidacloprid affected the mortality of fourth instar larvae of *C. septempunctata* 1, 3, 10 and 21 DAT (Figure 6). The mortality of fourth instar larvae of *C. septempunctata* was significantly increased 1 DAT (χ^2^ = 112.86, df = 2, *p* < 0.001), 3 DAT (χ^2^ = 145.12, df = 2, *p* < 0.001), 10 DAT (χ^2^ = 45.48, df = 2, *p* < 0.001) and 3 days after predator exposure to deltamethrin or imidacloprid residues on pepper plants compared to the control. No significant differences were observed at 21 DAT and 3 days after predator exposure to insecticides (χ^2^ = 2.01, df = 2, *p* = 0.366). Mortality was significantly higher when exposed for 7 days to insecticide residues at 1 DAT (χ^2^ = 121.25, df = 2, *p* < 0.001), 3 DAT (χ^2^ = 122.58, df = 2, *p* < 0.001), 10 DAT (χ^2^ = 132.36, df = 2, *p* < 0.001) and 21 DAT (χ^2^ = 138.47, df = 2, *p* < 0.001). *C. septempunctata* mortality was significantly higher when exposed to deltamethrin residues on pepper plants for 7 days than the control and the imidacloprid treatments. The mortality of *C. septempunctata* larvae by deltamethrin 3 days after larvae exposure was 100%, 98.33%, 41.67% and 0% at 1, 3, 10 and 21 DAT, respectively. The corresponding values for imidacloprid were 45%, 11.67%, 6.67% and 1.67%, respectively. In contrast, larvae mortality was 100% when exposed to deltamethrin residues on pepper plants for 7 days at 1, 3, 10 and 21 DAT. The corresponding values for imidacloprid were 75%, 25%, 20% and 18.33% at 1, 3, 10 and 21 DAT, respectively.

## 4. Discussion

Combining biological control and insecticide use in IPM programs requires information on how insecticides affect not only the target pest but also their natural enemies [27,28]. In the present study, the toxicity and the sublethal and residual effect of deltamethrin and imidacloprid on the seven-spot ladybeetle, *C. septempunctata,* were examined. Pyrethroid and neonicotinoid insecticides can have lethal and sublethal effects on coccinellid predators [28,29,30,31,32,33,34,35,36,37,38,39]. Deltamethrin and imidacloprid, which are frequently used insecticides in greenhouses to control aphids, have direct toxic effects on the fourth instar larvae of *C. septempunctata*, but among the two insecticides tested, deltamethrin was much more toxic than imidacloprid. Deltamethrin, a widely used pyrethroid, was found to be 685 times more toxic for the fourth instar larvae of *C. septempunctata* than imidacloprid. This difference in toxicity between the two insecticides was likely due to the resistance development in *C. septempunctata* to imidacloprid due to its frequent use to control *M. persicae* in tobacco and peach orchards for more than three decades in northern Greece [5,40], in addition to detoxification enzymes or activity in target site sensitivity by each insecticide [38]. The same toxic results to coccinellid predators by pyrethroids have been reported by many researchers [29,33,41] in *Adalia bipunctata* (L.) and *Ceratomegilla undecimnotata* (Schneider, 1792) [37]. Furthermore, residues of the insecticide deltamethrin were more toxic for the fourth instar larvae of *C. septempunctata* than those of imidacloprid. The mortality of the fourth instar larvae of *C. septempunctata* for imidacloprid began in the slightly harmful category (75%) 1 DAT and declined to the harmless category (18.33%) 21 DAT. In contrast, deltamethrin was placed in the harmful category (100%) during the entire examined period. Briefly, deltamethrin was toxic to biological control agents for aphid control in greenhouses, and the number of days for release after treatment should be carefully considered when using *C. septempunctata* in IPM programs.

A sublethal dose of deltamethrin and imidacloprid increased the developmental time of fourth instar larvae of *C. septempunctata*. Prolonged developmental time has been reported by several authors in *C. septempunctata* after fourth instar larvae are treated with sublethal doses of imidacloprid [42,43]. Moreover, increased developmental time was caused by imidacloprid in fourth instar larvae of *C. undecimnotata* and *Hippodamia variegata* (Goeze) (Coleoptera: Coccinellidae) [36,37]. Increased developmental time was caused by bifenthrin to the second instar larvae of *C. septempunctata* [35]. The prolonged developmental time compared to the control group may be due to the fact that deltamethrin- or imidacloprid-treated larvae groups used their energy to detoxify the insecticides rather than for their development [44] or/and decreased pest consumption and, as a result, reduced their energy supply [30,31,36].

Although the duration of fourth instar larvae was impressively increased by the sublethal doses (LD_10_ and LD_30_) of deltamethrin and imidacloprid, the adult total longevity decreased compared to the control group. Furthermore, fecundity was significantly reduced after treatment with the LD_10_ of imidacloprid and deltamethrin and the LD_30_ of imidacloprid. These results are similar to those of other studies, where imidacloprid and deltamethrin decreased the adult longevity and fecundity of coccinellid predators [36,37,39,45]. The decreased fecundity could be attributed to the reduced food intake at the fourth instar larvae of *C. septempunctata* due to the insecticide treatment, which affected adult fitness [38,46]. Decreased fecundity may be based on the direct toxic effect of the insecticide and/or malformations of organs [47]. In addition to fecundity, the developmental time and longevity, value of the intrinsic rate of increase (*r*), net reproductive rate (*R*_0_) and finite rate of increase (*λ*) can be useful for understanding the predator coccinellid population dynamics. Our results showed that the *r*, *λ* and *R*_0_ of *C. septempunctata* decreased under the sublethal doses of imidacloprid compared to the control group. Sublethal doses of deltamethrin decreased the *R*_0_ of *C. septempunctata* compared to the control group. The results indicate that both sublethal doses of imidacloprid and deltamethrin can produce detrimental effects on the physiology of *C. septempunctata* [34]. Sublethal doses of neonicotinoids have been reported to lower population growth parameters for many coccinellid predators and/or other insects [30,32,36,37,39,47].

Furthermore, in our study, we found that imidacloprid and deltamethrin sublethal doses affected the two-sex life table parameters of *C. septempunctata*. Values such as *m_x_*, *l_x_*, *l_x_m_x_*, *e_xj_*, *V_xj_* and *S_xj_* show a decreasing pattern, indicating that the life table parameters were affected by deltamethrin and imidacloprid at sublethal doses of LD_10_ and LD_30_. The reduction in the life table parameters such as *m_x_* and *l_x_* might be due to the fact that insecticides kill the more sensitive individuals, while the more resistant reduce prey consumption [32,38,46,48]. This result supports the idea that the reduced population parameters of the coccinellid predator verify that sublethal doses of deltamethrin and imidacloprid can reduce the survival and reproduction, thereby minimizing its efficacy as an aphid predator in greenhouse IPM strategies.

## 5. Conclusions

In conclusion, our study showed that the residual toxicity of *C. septempunctata* varied between imidacloprid and deltamethrin. Imidacloprid had lower residual toxicity than deltamethrin to fourth instar larvae of *C. septempunctata*. In the present laboratory experiments, deltamethrin and imidacloprid had negative effects on the population parameters and survival of *C. septempunctata*. These findings indicate that both insecticides should not be preferred for IPM programs of *M. persicae* and other aphids. In particular, deltamethrin should be avoided due to its extreme toxicity to fourth instar larvae of *C. septempunctata*, which lasts for up to three weeks after application. These data provide the basis for new studies on the residual toxicity of the insecticides tested on coccinellid predators beyond 21 DAT performed under greenhouse conditions.

## Figures and Tables

**Table 1:** Toxicity of imidacloprid and deltamethrin to fourth instars larvae of *Coccinella septempunctata* in lab bioassays after 72 h of treatment.

Insecticide	N ^a^	Dose Nanograms (a.i.) per Insect (95% CL)^−1^			Slope ± SE	χ^2^	*p*	df
		LD_10_	LD_30_	LD_50_				
Imidacloprid	540	357.96 (301.27–406.91)	519.13 (464.81–567.17)	671.56 (619.02–723.76)	4.690 ± 0.408	3.778	0.707	6
Deltamethrin	480	0.337 (0.260–0.413)	0.633 (0.530–0.737)	0.980 (0.848–1.127)	2.763 ± 0.215	1.687	0.891	5

^a^ Number of insects tested.

**Table 2:** Sublethal effects of imidacloprid and deltamethrin on the developmental time (mean ± SE) of *C. septempunctata* adults exposed to insecticide from the fourth instar larval stage.

Treatments	N ^a^	Development Time of Fourth Instar Larva (d)	N ^a^	Development Time of Pupa (d)
Control	35	4.51 ± 0.12 b	35	5.03 ± 0.10 a
Imidacloprid LD_10_	39	5.17 ± 0.18 a	29	4.96 ± 0.13 ab
Imidacloprid LD_30_	53	5.28 ± 0.15 a	29	5.04 ± 0.11 a
Deltamethrin LD_10_	43	5.18 ± 0.17 a	28	4.64 ± 0.11 b
Deltamethrin LD_30_	47	5.11 ± 0.17 a	28	4.82 ± 0.14 ab

Means followed by the same letters in the same column are not significantly different based on the paired bootstrap test at the 5% significance level. ^a^ Number of insects tested.

**Table 3:** Sublethal effects of imidacloprid and deltamethrin on the life parameters (mean ± SE) of *C. septempunctata* adults exposed to insecticide from the fourth instar larval stage.

Treatments	Adult Longevity (d)	N ^a^	Male Adult Longevity (d)	N ^a^	Female Adult Longevity (d)	APOP (d)	TPOP (d)	Fecundity (Eggs/Female)
Control	73.63 ± 2.34 a	18	70.11 ± 3.29 a	17	77.35 ± 3.17 a	8.88 ± 0.73 a	27.76 ± 0.72 a	758.12 ± 48.24 a
Imidacloprid LD_10_	63.38 ± 3.77 b	13	61.23 ± 6.42 ab	13	65.54 ± 4.14 b	13.85 ± 2.78 a	33.77 ± 2.83 a	363.38 ± 99.66 bc
Imidacloprid LD_30_	55.33 ± 5.27 b	12	50.33 ± 6.80 b	12	60.33 ± 8.07 b	13.58 ± 1.19 a	33.75 ± 1.46 a	228.25 ± 57.58 c
Deltamethrin LD_10_	58.82 ± 4.33 b	14	50.79 ± 5.49 b	14	66.86 ± 6.44 ab	12.64 ± 2.75 a	30.71 ± 2.72 a	322.00 ± 60.06 bc
Deltamethrin LD_30_	59.43 ± 4.71 b	15	52.67 ± 5.85 b	13	67.23 ± 7.21 ab	9.00 ± 2.03 a	28.08 ± 2.13 a	517.62 ± 132.81 ab

Means followed by the same letters in the same column are not significantly different based on the paired bootstrap test at the 5% significance level. ^a^ Number of insects tested.

**Table 4:** Sublethal effects of imidacloprid and deltamethrin on the population growth parameters (mean ± SE) of *C. septempunctata* adults exposed to insecticide from the fourth instar larval stage.

Treatments	N ^a^	Intrinsic Rate of Increase (*r*) Day^−1^	Net Reproductive Rate (*R*_0_) (Offspring per Individual)	Mean Generation Time (*T*) (Days)	Finite Rate of Increase (*λ*) (Day^−1^)
Control	65	0.121164 ± 0.006 a	198.28 ± 43.07 a	42.66 ± 0.99 a	1.12 ± 0.007 a
Imidacloprid LD_10_	71	0.086798± 0.009 b	66.54 ± 24.19 b	48.36 ± 4.27 a	1.09 ± 0.010 b
Imidacloprid LD_30_	80	0.077889 ± 0.012 b	34.24 ± 12.31 b	45.36 ± 5.27 a	1.08 ± 0.013 b
Deltamethrin LD_10_	70	0.105859 ± 0.013 ab	64.40 ± 19.30 b	39.35 ± 3.67 a	1.11 ± 0.015 ab
Deltamethrin LD_30_	75	0.104097 ± 0.012 ab	89.72 ± 31.67 b	43.20 ± 3.53 a	1.11 ± 0.013 ab

Means followed by the same letters in the same column are not significantly different based on the paired bootstrap test at the 5% significance level. ^a^ Number of insects tested.

## Data Availability

Available upon request.

## Supplementary Materials

The following supporting information can be downloaded at: https://www.mdpi.com/article/10.3390/toxics11070584/s1, Figure S1: Variation of temperature and relative humidity in greenhouse during the assessing the residual toxicity of imidacloprid and deltamethrin of *C. septempunctata*.

## References

[1] Pineda A., Angeles Marcos-Garcia M.J.E. (2008). Introducing barley as aphid reservoir in sweet-pepper greenhouses: Effects on native and released hoverflies (Diptera: Syrphidae). Eur. J. Entomol..

[2] Blackman R.L., Eastop V.F., Van Emden H.F., Harrington R. (2017). Taxonomic Issues. Aphids as Crop Pests.

[3] Goggin F.L. (2007). Plant–aphid interactions: Molecular and ecological perspectives. Curr. Opin. Plant Biol..

[4] Bass C., Puinean A.M., Zimmer C.T., Denholm I., Field L.M., Foster S.P., Gutbrod O., Nauen R., Slater R., Williamson M.S. (2014). The evolution of insecticide resistance in the peach potato aphid, *Myzus persicae*. Insect Biochem. Mol. Biol..

[5] Margaritopoulos J.T., Kati A.N., Voudouris C.C., Skouras P.J., Tsitsipis J.A. (2021). Long-term studies on the evolution of resistance of *Myzus persicae* (Hemiptera: Aphididae) to insecticides in Greece. Bull. Entomol. Res..

[6] Singh K.S., Cordeiro E.M.G., Troczka B.J., Pym A., Mackisack J., Mathers T.C., Duarte A., Legeai F., Robin S., Bielza P. (2021). Global patterns in genomic diversity underpinning the evolution of insecticide resistance in the aphid crop pest *Myzus persicae*. Commun. Biol..

[7] Umina P.A., Bass C., van Rooyen A., Chirgwin E., Arthur A.L., Pym A., Mackisack J., Mathews A., Kirkland L. (2022). Spi-rotetramat resistance in *Myzus persicae* (Sulzer) (Hemiptera: Aphididae) and its association with the presence of the A2666V mutation. Pest Manag. Sci..

[8] Overton K., Ward S.E., Hoffmann A.A., Umina P.A. (2023). Lethal impacts of insecticides and miticides on three agriculturally important aphid parasitoids. Biol. Control.

[9] Riddick E.W. (2017). Identification of Conditions for Successful Aphid Control by Ladybirds in Greenhouses. Insects.

[10] Powell W., Pell J.K., Van Emden H.F., Harrington R. (2017). Biological Control. Aphids as Crop Pests.

[11] Van Driesche R.G., van Driesche R., Heinz K.M., Parrella M.P. (2004). An overview of biological control in protected culture. BioControl in Protected Culture.

[12] Yano E. (2006). Ecological considerations for biological control of aphids in protected culture. Popul. Ecol..

[13] Hodek I., Honek A., Van Emden H.F. (2012). Ecology and Behaviour of the Ladybird Beetles (Coccinellidae).

[14] Singh S.R., Walters K.F.A., Port G.R., Northing P. (2004). Consumption rates and predatory activity of adult and fourth instar larvae of the seven spot ladybird, *Coccinella septempunctata* (L.), following contact with dimethoate residue and contam-inated prey in laboratory arenas. Biol. Control.

[15] Zhang P., Lu Y., Chao W., Dong Z., Ali A., Liu T.X., Lu Z. (2022). When Does the Prey/Predator Ratio Work for the Effective Biocontrol of Cotton Aphid on Cotton Seedlings?. Insects.

[16] Bass C., Denholm I., Williamson M.S., Nauen R. (2015). The global status of insect resistance to neonicotinoid insecticides. Pestic. Biochem. Physiol..

[17] Authority E.F.S. (2013). Guidance on the risk assessment of plant protection products on bees (*Apis mellifera*, *Bombus* spp. and solitary bees). EFSA J..

[18] Tasman K., Hidalgo S., Zhu B., Rands S.A., Hodge J.J.L. (2021). Neonicotinoids disrupt memory, circadian behaviour and sleep. Sci. Rep..

[19] Santos A.C.C., Cristaldo P.F., Araújo A.P.A., Melo C.R., Lima A.P.S., Santana E.D.R., de Oliveira B.M.S., Oliveira J.W.S., Vieira J.S., Blank A.F. (2018). Apis mellifera (Insecta: Hymenoptera) in the target of neonicotinoids: A one-way ticket? Bi-oinsecticides can be an alternative. Ecotoxicol. Environ. Saf..

[20] Medina P., Budia F., Estal P.D., Adán A., Viñuela E. (2004). Toxicity of Fipronil to the Predatory Lacewing *Chrysoperla carnea* (Neuroptera: Chrysopidae). Biocontrol Sci. Technol..

[21] Stark J.D., Jepson P.C., Mayer D.F. (1995). Limitations to Use of Topical Toxicity Data for Predictions of Pesticide Side Effects in the Field. J. Econ. Entomol..

[22] Blackman R.L. (1971). Variation in the photoperiodic response within natural populations of *Myzus persicae* (Sulz.). Bull. Entomol. Res..

[23] Chi H. (2021). TWOSEX-MSChart: A Computer Program for the Age-Stage, Two-Sex Life Table Analysis.

[24] Chi H. (1988). Life-Table Analysis Incorporating Both Sexes and Variable Development Rates Among Individuals. Environ. Entomol..

[25] Chi H., Liu H. (1985). Two new methods for the study of insect population ecology. Bull. Inst. Zool. Acad. Sin..

[26] Wei M., Chi H., Guo Y., Li X., Zhao L., Ma R. (2020). Demography of *Cacopsylla chinensis* (Hemiptera: Psyllidae) Reared on Four Cultivars of *Pyrus bretschneideri* (Rosales: Rosaceae) and *P. communis* Pears with Estimations of Confidence Intervals of Specific Life Table Statistics. J. Econ. Entomol..

[27] Desneux N., Decourtye A., Delpuech J.-M. (2007). The Sublethal Effects of Pesticides on Beneficial Arthropods. Annu. Rev. Entomol..

[28] Quesada C.R., Sadof C.S. (2020). Residual toxicity of insecticides to Chrysoperla rufilabris and Rhyzobius lophanthae predators as biocontrol agents of pine needle scale. Crop Prot..

[29] Garzón A., Medina P., Amor F., Viñuela E., Budia F. (2015). Toxicity and sublethal effects of six insecticides to last instar larvae and adults of the biocontrol agents *Chrysoperla carnea* (Stephens) (Neuroptera: Chrysopidae) and *Adalia bipunctata* (L.) (Coleoptera: Coccinellidae). Chemosphere.

[30] He F., Sun S., Tan H., Sun X., Shang D., Yao C., Qin C., Ji S., Li X., Zhang J. (2019). Compatibility of chlorantraniliprole with the generalist predator *Coccinella septempunctata* L. (Coleoptera: Coccinellidae) based toxicity, life-cycle development and population parameters in laboratory microcosms. Chemosphere.

[31] He Y., Zhao J., Zheng Y., Desneux N., Wu K. (2012). Lethal effect of imidacloprid on the coccinellid predator Serangium japonicum and sublethal effects on predator voracity and on functional response to the whitefly Bemisia tabaci. Ecotoxicology.

[32] Jiang J., Zhang Z., Yu X., Yu C., Liu F., Mu W. (2019). Sublethal and transgenerational effects of thiamethoxam on the demo-graphic fitness and predation performance of the seven-spot ladybeetle *Coccinella septempunctata* L. (Coleoptera: Coc-cinellidae). Chemosphere.

[33] Olszak R.W. (1999). Influence of some pesticides on mortality and fecundity of the aphidophagous coccinellid *Adalia bipunctata* L. (Col., Coccinellidae). J. Appl. Entomol..

[34] Rahmani S., Bandani A.R. (2013). Sublethal concentrations of thiamethoxam adversely affect life table parameters of the aphid predator, *Hippodamia variegata* (Goeze) (Coleoptera: Coccinellidae). Crop Prot..

[35] Rizwan M., Atta B., Rizwan M., Ashraf I., Arshad M., Tahir M., Ali M., Sabir A.M., Bilal M., Ali M.Y. (2021). Do neonicotinoids better than pyrethroids for *Coccinella septempunctata* L. (Coleoptera: Coccinellidae)? A comparative sub-lethal indirect age-stage, two-sex life tables laboratory bioassay. Int. J. Trop. Insect Sci..

[36] Skouras P.J., Brokaki M., Stathas G.J., Demopoulos V., Louloudakis G., Margaritopoulos J.T. (2019). Lethal and sub-lethal effects of imidacloprid on the aphidophagous coccinellid *Hippodamia variegata*. Chemosphere.

[37] Skouras P.J., Darras A.I., Mprokaki M., Demopoulos V., Margaritopoulos J.T., Delis C., Stathas G.J. (2021). Toxicity, sublethal and low dose effects of imidacloprid and deltamethrin on the aphidophagous predator *Ceratomegilla undecimnotata* (Coleoptera: Coccinellidae). Insects.

[38] Wang L., Zhai Y., Zhu J., Wang Q., Ji X., Wang W., Yuan H., Rui C., Cui L. (2023). Sulfoxaflor adversely influences the bio-logical characteristics of *Coccinella septempunctata* by suppressing vitellogenin expression and predation activity. J. Hazard. Mater..

[39] Xiao D., Zhao J., Guo X., Chen H., Qu M., Zhai W., Desneux N., Biondi A., Zhang F., Wang S. (2016). Sublethal effects of imidacloprid on the predatory seven-spot ladybird beetle *Coccinella septempunctata*. Ecotoxicology.

[40] Margaritopoulos J.T., Skouras P.J., Nikolaidou P., Manolikaki J., Maritsa K., Tsamandani K., Kanavaki O.M., Bacan-dritsos N., Zarpas K.D., Tsitsipis J.A. (2007). Insecticide resistance status of *Myzus persicae* (Hemiptera: Aphididae) populations from peach and tobacco in mainland Greece. Pest Manag. Sci..

[41] Jalali M.A., Van Leeuwen T., Tirry L., De Clercq P. (2009). Toxicity of selected insecticides to the two-spot ladybird *Adalia bipunctata*. Phytoparasitica.

[42] Afza R., Afzal A., Riaz M.A., Majeed M.Z., Idrees A., Qadir Z.A., Afzal M., Hassan B., Li J. (2023). Sublethal and transgener-ational effects of synthetic insecticides on the biological parameters and functional response of *Coccinella septempunctata* (Coleoptera: Coccinellidae) under laboratory conditions. Front. Physiol..

[43] Skouras P.J., Stathas G.J., Voudouris C.C., Darras A.I., Tsitsipis J.A., Margaritopoulos J.T. (2017). Effect of synthetic insecticides on the larvae of *Coccinella septempunctata* from Greek populations. Phytoparasitica.

[44] Hannig G.T., Ziegler M., Marçon P.G. (2009). Feeding cessation effects of chlorantraniliprole, a new anthranilic diamide in-secticide, in comparison with several insecticides in distinct chemical classes and mode-of-action groups. Pest Manag. Sci..

[45] Yu C., Lin R., Fu M., Zhou Y., Zong F., Jiang H., Lv N., Piao X., Zhang J., Liu Y. (2014). Impact of imidacloprid on life-cycle development of *Coccinella septempunctata* in laboratory microcosms. Ecotoxicol. Environ. Saf..

[46] Xie J., De Clercq P., Pan C., Li H., Zhang Y., Pang H. (2015). Larval nutrition-induced plasticity affects reproduction and gene expression of the ladybeetle, Cryptolaemus montrouzieri. BMC Evol. Biol..

[47] Ju D., Liu Y.-X., Liu X., Dewer Y., Mota-Sanchez D., Yang X.-Q. (2023). Exposure to lambda-cyhalothrin and abamectin drives sublethal and transgenerational effects on the development and reproduction of *Cydia pomonella*. Ecotoxicol. Environ. Saf..

[48] Xu C., Zhang Z., Cui K., Zhao Y., Han J., Liu F., Mu W. (2016). Effects of Sublethal Concentrations of Cyantraniliprole on the Development, Fecundity and Nutritional Physiology of the Black Cutworm *Agrotis ipsilon* (Lepidoptera: Noctuidae). PLoS ONE.

