# Supplementary material for: Toxicity and Lethal Effect of Greenhouse Insecticides on Coccinella septempunctata (Coleoptera: Coccinellidae) as Biological Control Agent of Myzus persicae (Hemiptera: Aphididae)
Source: Toxics. 2023 Jul 5;11(7):584. doi: 10.3390/toxics11070584 (PMC10385029; doi:10.3390/toxics11070584)
Supplement: Supplementary file 1 [file toxics-11-00584-s001.zip › toxics-2408150-supplementary.pdf]

Toxicity and Lethal Effect of Greenhouse Insecticides on *Coccinella septempunctata* (Coleoptera: Coccinellidae) as Biological Control Agent of *Myzus persicae* (Hemiptera: Aphididae)

Panagiotis J. Skouras [1,2,*], Eirini Karanastasi [3], Giannis Lykoskoufis [4], Vasilis Demopoulos [2], Anastasios Darras [4], Athanasios Tsafouros [4], Polina C. Tsalgatidou [4], John T. Margaritopoulos [5] and George J. Stathas [1]

1 Laboratory of Agricultural Entomology and Zoology, Department of Agriculture,
University of the Peloponnese, Kalamata Campus, 24100 Antikalamos, Greece; g.stathas@uop.gr

2 Laboratory of Plant Protection, Department of Agriculture, University of the Peloponnese,
Kalamata Campus, 24100 Antikalamos, Greece; v.dimopoulos@go.uop.gr

3 Plant Protection Laboratory, Department of Agriculture, University of Patras, Nea Ktiria,
30200 Mesolonghi, Greece; ekaranastasi@upatras.gr

4 Department of Agriculture, University of the Peloponnese, 24100 Kalamata, Greece;
i.lykoskoufis@uop.gr (G.L.); a.darras@uop.gr (A.D.); thantsaf@hotmail.com (A.T.);
polina.tsalgatidou@go.uop.gr (P.C.T.)

5 Department of Plant Protection, Institute of Industrial and Fodder Crops, Hellenic Agricultural Organization "DEMETER", 38334 Volos, Greece; johnmargaritopoulos@elgo.com

* Correspondence: p.skouras@go.uop.gr or pskouras@windowslive.com; Tel.: +30-(27)-21045277;
Fax: +30-(27)-21045234

The experiment was conducted in a typical Venlo-type greenhouse with an area of 200m^2, covered with polyethylene and located at the experimental field of the Agriculture Department of the University of Peloponnese, Kalamata is 5m above sea level (latitude 37°03′40″ N, longitude 22°03′41″ E). The greenhouse air temperature was not left to fall below 16°C by using a fan heater.

The greenhouse air temperature was measured with copper–constantan thermocouples (0.2mm), which were placed in a ventilated chamber in the center of the greenhouse and at a height of 1.5m from the soil. A relative humidity sensor (Thermometriks, Na1015) was placed in the same ventilated chamber. Solar radiation inside the greenhouse was measured with a

pyranometer (CM 11, Kipp & Zonen, The Netherlands). Every 10 minutes, the values of the above measurements were recorded by a datalogger (DL 2e, Delta-T Devices, UK).

The night-period greenhouse air temperature fluctuated between 15.8 and 18.9°C, while the day-period greenhouse temperature fluctuated between 17.1 and 29.6°C. The 24-hour mean temperature was 19.8°C. Relative humidity during the night ranged between 67 and 90%, and during the day, it ranged between 48 and 90% (Fig S1). The mean incident solar radiation in the greenhouse during the day was 193W m^{-2} .

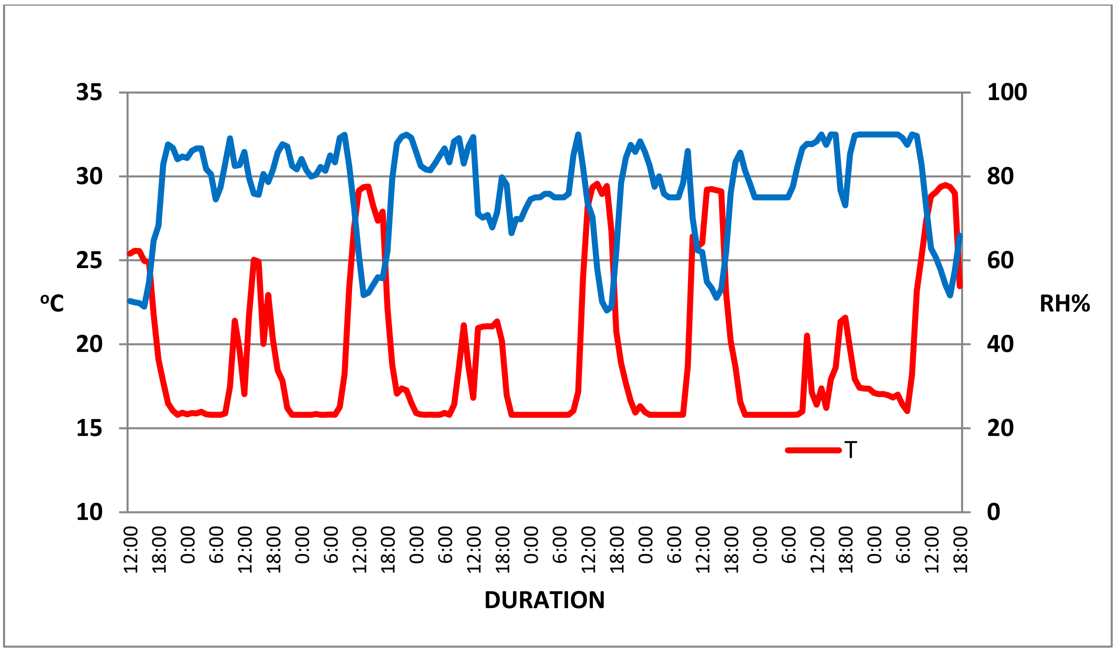

Fig S1. Variation of temperature and relative humidity in greenhouse during the assessing the residual toxicity of imidacloprid and deltamethrin of *C. septempunctata*.
